# Radiomics model based on contrast-enhanced CT texture features for pretreatment prediction of overall survival in esophageal neuroendocrine carcinoma

**DOI:** 10.3389/fonc.2023.1225180

**Published:** 2023-08-18

**Authors:** Yue Zhou, Lijie Song, Jin Xia, Huan Liu, Jingjing Xing, Jianbo Gao

**Affiliations:** ^1^ Department of Radiology, First Affiliated Hospital of Zhengzhou University, Zhengzhou, China; ^2^ Department of Oncology, First Affiliated Hospital of Zhengzhou University, Zhengzhou, China; ^3^ Department of Oncology, Anyang Tumor Hospital, Anyang, China; ^4^ Advanced Analytics Team, GE Healthcare, Shanghai, China

**Keywords:** esophageal neoplasm, tomography, radiomics, nomogram, survival

## Abstract

**Background:**

Limited studies have observed the prognostic value of CT images for esophageal neuroendocrine carcinoma (NEC) due to rare incidence and low treatment experience in clinical. In this study, the pretreatment enhanced CT texture features and clinical characteristics were investigated to predict the overall survival of esophageal NEC.

**Methods:**

This retrospective study included 89 patients with esophageal NEC. The training and testing cohorts comprised 61 (70%) and 28 (30%) patients, respectively. A total of 402 radiomics features were extracted from the tumor region that segmented pretreatment venous phase CT images. The least absolute shrinkage and selection operator (LASSO) Cox regression was applied to feature dimension reduction, feature selection, and radiomics signature construction. A radiomics nomogram was constructed based on the radiomics signature and clinical risk factors using a multivariable Cox proportional regression. The performance of the nomogram for the pretreatment prediction of overall survival (OS) was evaluated for discrimination and calibration.

**Results:**

Only the enhancement degree was an independent factor in clinical variable influenced OS. The radiomics signatures demonstrated good predictability for prognostic status discrimination. The radiomics nomogram integrating texture signatures was slightly superior to the nomogram derived from the combined model with a C-index of 0.844 (95%CI: 0.783-0.905) and 0.847 (95% CI: 0.782-0.912) in the training set, and 0.805 (95%CI: 0.707-0.903) and 0.745 (95% CI: 0.639-0.851) in the testing set, respectively.

**Conclusion:**

The radiomics nomogram based on pretreatment CT radiomics signature had better prognostic power and predictability of the overall survival in patients with esophageal NEC than the model using combined variables.

## 1 Introduction

Neuroendocrine carcinoma (NEC) is a malignant tumor originating from the neuroendocrine cells, and most commonly occurs in the lungs and the digestive system (1). Esophageal NEC is a rare disease with a reported incidence of between 0.05% and 5.9% among all malignancies affecting the esophagus (2). At the time of diagnosis, the disease displays aggressive progression with poor prognosis because the neuroendocrine system is not well developed in the esophagus (1). Unlike esophageal squamous cell carcinoma (ESCC) and esophageal adenocarcinoma (EAC), NEC can be diagnosed and classified using a grading system based on mitotic count and the Ki-67 proliferation index (3). Esophageal NEC is entirely defined by high grade ratings (G3) and high ratios of Ki-67 (2–5). Surgery combined with chemoradiotherapy is considered the most ideal treatment strategy for esophageal NEC due to its highly invasive nature and tendency for early metastasis (6). However, a rare incidence and low treatment experience result in insufficient clinical observations and suitable guidelines for the management of esophageal NEC.

It has been proven that angiogenic CT parameters play a predictive role for tumor progression and prognosis after surgery or neoadjuvant chemotherapy/chemoradiotherapy (7, 8). A few retrospective clinical esophageal NEC analyses have shown better survival effects with a combination of radical resection, radical lymph node dissection, and adjuvant therapy (9–11). However, the survival investigations involving esophageal NEC are based on basic clinical prognostic evaluation. Studies involving evaluation of tumor heterogeneity and therapeutic efficacy have not been conducted yet.

Radiomics has increasingly emerged as a technique for improved tumor evaluation, whereby a huge amount of data may be extracted from medical images through post-processing techniques, thus enabling quantification of the heterogeneity within the tumor (12). Assessment of the tumor texture on images of esophageal cancers has been performed earlier and shown to carry prognostic information in terms of predicting survival (13, 14). Recently, Riyahi et al. demonstrated feature extraction of CT images of patients with locally advanced esophageal cancer before chemoradiotherapy and generated a sensitivity and specificity of 94.4% and 91.8%, respectively (15). To the best of our knowledge, the potential of CT texture analysis as a therapeutic responder or as a predictor of treatment efficacy in esophageal NEC has never been investigated.

The aim of this study was to investigate the role of pretreatment CT imaging radiomics signatures using texture features, and that of clinical characteristics in predicting the overall survival of patients with esophageal NEC.

## 2 Materials and methods

### 2.1 Subject enrollment

This study was approved by the Institutional Review Board (IRB) of the First Affiliated Hospital of Zhengzhou University. All patients enrolled in this study provided informed consent.

A total of 115 patients who had a confirmatory diagnosis of esophageal NEC during the period from September 2015 to July 2019 were included in our database. We confirmed the diagnosis of esophageal NEC, without any squamous carcinoma/adenocarcinoma component mixed, on the basis of pathological and immunohistochemical specimen via esophagoscope before surgery or intraoperatively. Patients who had earlier undergone a baseline contrast-enhanced CT before treatment in our institution were enrolled in the study. The remaining 89 patients (median age, 65 years; range, 42-79 years) comprising of 57 males and 32 females were included in the study for treatment observation and subsequent follow-up investigation.

Of the 89 patients, 61 patients (70%) (median age, 65 years; range, 42-78 years) were included in a training cohort for exploring the independent predictors of the texture parameters as well as to optimize the threshold in differentiating the patients’ survival outcomes. The remaining 28 patients (30%) (median age, 65 years; range, 45-79 years) comprised a testing cohort, which was used to validate the prognostic significance of the identified predictors, by applying optimal cutoff values in an external verification set.

### 2.2 Treatment

The treatment strategies for NEC included esophagectomy (n = 5), chemotherapy (n = 40), radiotherapy (n = 1), chemoradiotherapy (n = 2), preoperative neoadjuvant chemotherapy (n = 1), and postoperative adjuvant chemotherapy (n = 39). The surgical approach for the 5 cases (6%) selected was based on the site of the lesion. The left thoracic approach into the chest was used for a lower esophageal lesion (n = 4), and a right thoracic approach was employed for an upper esophageal lesion (n = 1). The treatment regimen for chemotherapy, preoperative neoadjuvant chemotherapy, and postoperative adjuvant chemotherapy included etoposide (Vepeside, VP - 16) 100mg/m^2^ IV on day 1 to day 5 and cisplatin (DDP) 75mg/m^2^ IV on the first day of a 21 - day cycle for a total of 4 to 6 cycles. Patients treated with radiotherapy received a dose of 1.8 Gray per day on day 1 to day 5, with total dose of 60 Gray at 33 days. Chemoradiotherapy regimen consisted of VP - 16, 50 mg/m^2^ and DDP, 20 mg/m^2^ on days 1 to 5, and a total of 60 Gray of concurrent radiotherapy as mentioned previously.

### 2.3 Patient follow-up

Therapeutic efficacy was evaluated 12 weeks after surgery by a CT enhancement examination. Patients who were treated with chemotherapy and/or radiotherapy also underwent a CT enhancement examination after every 3 cycles from the beginning of treatment for the first 12 cycles and at every 4 cycles thereafter. All patients underwent clinical follow up through an outpatient service or a telephone communication (every 12 weeks) until death or the end of the study. The OS was set (time from the date of diagnosis to death from any cause) as the main endpoint and was predicted by constructing nomograms.

### 2.4 CT image acquisition

A baseline CT scan was obtained less than a week before treatment was initiated. All patients fasted for 6 hours. 20 mg of amidoamine was injected intramuscularly 10 - 15 minutes before CT examination. In order to distend the upper gastrointestinal tract, the subjects were first asked to drink 800 - 1000ml water, and subsequently advised to drink 200 - 300 ml water with a straw in a supine position. A contrast-enhanced CT scan of the chest was performed with a 64 - slice CT scanner (Lightspeed VCT, GE Healthcare, Waukesha, WI, USA) or a 256 - slice CT scanner (Revolution CT, GE Healthcare, Waukesha, WI, USA). The scan mode settings comprised of a tube voltage of 120 kV or 80 kV/140 kV with a fast kV - switching technique, tube current under Auto mA with a noise index of 8.0, a slice thickness of 5 mm, a gantry speed of 0.6 seconds per rotation and a pitch of 0.984: 1. A contrast medium containing 350 mg of iodine per ml (Omnipaque™; GE Healthcare, Cork, Ireland) was injected at a flow rate of 3 ml/s via the elbow vein. The dose of the contrast medium was calculated as 1.5 ml per kg body weight. Scanning was triggered when the CT value of the aortic arch reached 100 HU. The venous phase was initiated 60 s after triggering the scan.

### 2.5 Clinical features selection

The basic morphological image features and clinical data assessed included the age and gender of the patient, tumor length, location, the thickness of esophageal tumor wall (Median), clinical symptoms, immunohistochemical index of markers such as Cytokeratin (CK), Chromogranin A (CgA), Synaptophysin (Syn), Neuron-specific enolase (NSE), and Ki-67 (%). Tumor invasion depth (cT stage), lymph node metastasis (cN stage), distant metastasis (cM stage), treatment strategy, tumor margin (well-defined or ill-defined), presence or absence of tumor calcification, morphological subtype and tumor homogeneity (homogeneous or presence of necrosis) were also assessed. The degree of enhancement was classified as slight, moderate, and marked enhancement, with the standard used for assessment of enhancement being: slight enhanced, the enhancement of the nodule was close to that of adjacent muscles; moderate enhanced, enhancement slightly higher than the adjacent muscles; marked enhanced, enhancement markedly higher than in the muscles (14). The start date of treatment, and the date of pretreatment CT examination were also documented.

### 2.6 Imaging segmentation

A radiologist (Y.Z.) with 12 years of experience in chest and abdominal CT diagnosis, independently retrieved the CT images in a DICOM format from the picture archiving and communication system (PACS), and resampled them to 1 × 1 × 1 mm^3^ sizes for maintaining a consistent thickness. The resampling images were subsequently loaded into the ITK-SNAP software (version 3.6.0; www.itksnap.org) for manual segmentation. Tumor volume of interest (VOI) segmentation was performed, and the images were exported in an NII. format for subsequent 3D reconstruction, data extraction, and analysis. First, the VOI was delineated to encompass the whole enhanced esophageal lesion in the sagittal position. Visually identified air density within the tumor and the normal esophageal wall adjacent to the margin of the lesion were excluded. Next, the outlined segmentation was compared and modified in an axial position and coronal position, respectively. A 3D reconstruction was precisely performed along the edge of the VOI region which was automatically separated from the other structures and the background.

### 2.7 Radiomics features extraction

The textural features were derived from a multiparadigm numerical computing and programming software Artificial Intelligence Kit (version 3.2.2, GE Healthcare). A total of 402 radiomics features were extracted for tumor characteristics from the VOI region segmented from the pretreatment venous phase CT images. The features were composed of the histogram (42 features), gray level co-occurrence matrix (GLCM) (144 features), gray level size zone matrix (GLSZM) (11 features), gray level run length matrix (GLRLM) (180 features), formfactor (15 features), and Haralick (10 features) (16–21). An analysis of variance (ANOVA) was performed for dimension reduction, and a correlation analysis was performed to reduce data redundancy. The details of features extraction are described in the **supplementary data**. The extracted texture features were standardized so as to remove the unit limits of the data of each feature.

A random selection of 30 enhanced CT images of the patients was selected for assessment of intraobserver agreement (20). The intraclass correlation coefficients (ICCs) were calculated to evaluate inter-observer variability of radiomics features and were classified as poor (< 0.40), fair (0.40 - 0.59), good (0.60 - 0.74), or excellent (0.75-1.00) (22). Highly correlated features (ICC ≥ 0.90) were identified and selected initially. After a spearman correlation analysis between the texture features, there were a remainder of 50 features.

### 2.8 Feature selection and radiomics signature building

Potential prognostic factors of survival were evaluated using a univariate Cox proportional hazard model based on forward stepwise selection from the clinical variables. A least absolute shrinkage and selection operator (LASSO) Cox regression method was used to determine features that predicted the overall survival. The radiomics score for each patient was computed using an equation in which the coefficients were derived from the LASSO Cox model. The radiomics score (Rad-score) was computed for each patient through a linear combination of selected features weighted by their respective coefficients. Feature selection and the radiomics signature building were performed in training and testing sets. A five-fold cross-validation test was used to detect the goodness of fit of the model. The model was validated for its calibration ability by calculating the probability of outcome for each patient of the whole dataset according to the model and comparing it with the actual survival of the patient. More details of LASSO Cox model and radiomics signature building are described in the **Supplementary Data**. The survival models used different sets of factors that included the radiomics score only, and a combined score using both selected radiomics features and clinical variables.

### 2.9 Statistical analysis

Statistical analysis was conducted with R software (version 3.4.4; http://www.Rproject.org) and *p* < 0.05 was considered statistically significant. The differences between the continuous variables were compared using the Mann-Whitney U test, and the differences between the categorical variables were analyzed by the Chi-square test. The predictive accuracy of the radiomics signature was quantified by receiver operator characteristic (ROC) curve in both, the training and the testing sets. The median value of each score in the model was used to stratify the patients into high-risk and low-risk groups (23). The potential correlation of the two models with the overall survival (OS) was assessed using Kaplan-Meier analysis. The difference in survival between the groups was calculated as hazard ratio (HR) for each model by use of log-rank tests.

## 3 Results

### 3.1 Patient characteristics

**Figure 1:**
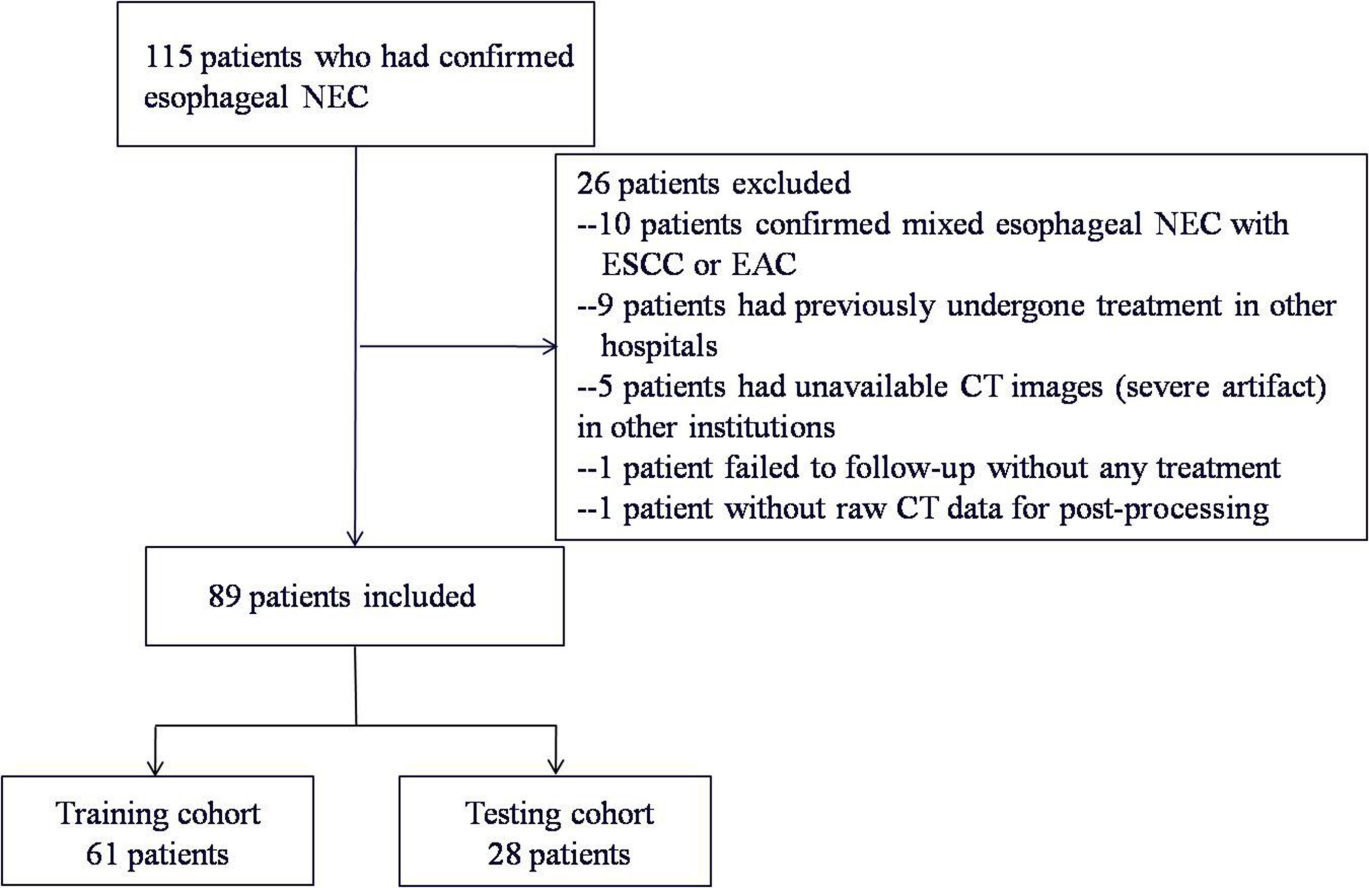
Flowchart describing study recruitment and enrollment process. NEC, neuroendocrine carcinoma; ESCC, esophageal squamous cell carcinoma; EAC, esophageal adenocarcinoma.

The details of patient enrollment are summarized in (**Figure 1**). The basic clinical characteristics of patients in the training and testing cohorts are shown in (**Table 1**). There was no statistical difference between the two cohorts in terms of the variables gender, age, morphological subtype, tumor length, location, clinical symptom, immunohistochemical result, the thickness of esophageal tumor wall, Ki-67 (%), cTNM stage, treatment strategy, tumor margin, necrosis, calcification, enhanced homogeneity, enhanced pattern, and enhanced degree. The median OS in the training and testing cohorts were 41.7 months (range: 33.4 - 50 months) and 45 months (range: 24.6 - 65.4 months), respectively (*p* = 0.302). OS was censored in 18 and 6 patients from the training and testing cohorts, respectively.

**Table 1:** Basic clinical characteristics of patients in the training and testing cohorts.

Variable	Training cohort (n=61)	Testing cohort (n=28)	*P*
Gender			0.325
Male	37 (61)	20 (71)	
Female	24 (39)	8 (29)	
Median age (years)*	65 (42-78)	65 (45-79)	0.56
Morphological subtype			0.282
Medullary type	20 (33)	12 (43)	
Mushroom type	13 (21)	2 (7)	
Ulcer type	10 (16)	3 (11)	
Narrowing type	18 (30)	11 (39)	
Median tumor length (mm)*	39.9 (1-99.7)	35.3 (12.2-120)	0.658
Tumor location			0.621
Upper segment esophagus	5 (8)	4 (14)	
Middle segment esophagus	37 (61)	17 (61)	
Lower segment esophagus	19 (31)	7 (25)	
Clinical symptom			0.94
Progressive dysphagia	38 (62)	19 (68)	
Abdominal/Retrosternal pain	12 (20)	6 (21)	
Loss of appetite	9 (15)	2 (7)	
Fever	2 (3)	1 (4)	
Immunohistochemical result			
CK	41 (67)	23 (82)	0.146
CgA	19 (31)	5 (18)	0.19
Syn	50 (82)	25 (89)	0.379
NSE	11 (18)	4 (14)	0.661
Ki-67 (Median, %)*	80 (60-100)	78 (60-95)	0.945
Median thickness of esophageal tumor wall (mm) *	10.9 (2-46)	11.1 (3-26.1)	0.356
T stage			0.313
T1-2	27 (44)	14 (50)	
T3	32 (53)	14 (50)	
T4	2 (3)	0	
N stage			0.77
Absent	22 (36)	11 (39)	
Present	39 (64)	17 (61)	
M stage			0.906
Absent	40 (66)	18 (64)	
Present	21 (34)	10 (36)	
Treatment strategy			0.344
Esophagectomy	3 (5)	2 (7)	
Chemotherapy only	30 (49)	11 (39)	
Radiotherapy only	0	1 (4)	
Neoadjuvant chemotherapy	1 (2)	2 (7)	
Postoperative adjuvant CRT	27 (44)	12 (43)	
Tumor margin			0.916
Well-defined	54 (89)	25 (89)	
Ill-defined	7 (11)	3 (11)	
Calcification			0.332
Absent	59 (97)	28 (100)	
Present	2 (3)	0	
Enhanced homogeneity			0.308
Homogeneous	55 (90)	27 (96)	
Presence of necrosis	6 (10)	1 (4)	
Enhanced degree			0.134
Slight enhancement	12 (20)	9 (32)	
Moderate enhancement	23 (37)	13 (46)	
Marked enhancement	26 (43)	6 (21)	
Median Hounsfield Units (HU) *	67.5 (40.8-96.2)	66.2 (43.1-87.3)	0.833
Median survival end points			
Overall survival(mon) *	41.7 (33.4-50)	45 (24.6-65.4)	0.302

Except where indicated, data are numbers of patients, with percentages in parentheses.

CK Cytokeratin, CgAchromogranin A, Syn synaptophysin, NSE neuron-specific enolase, CRT Chemoradiotherapy, mm Millimeter, HU Hounsfield unit, mon month.

* Numbers in parentheses are ranges.

### 3.2 Intra-observer reproducibility analysis and radiomics features extraction

**Figure 2:**
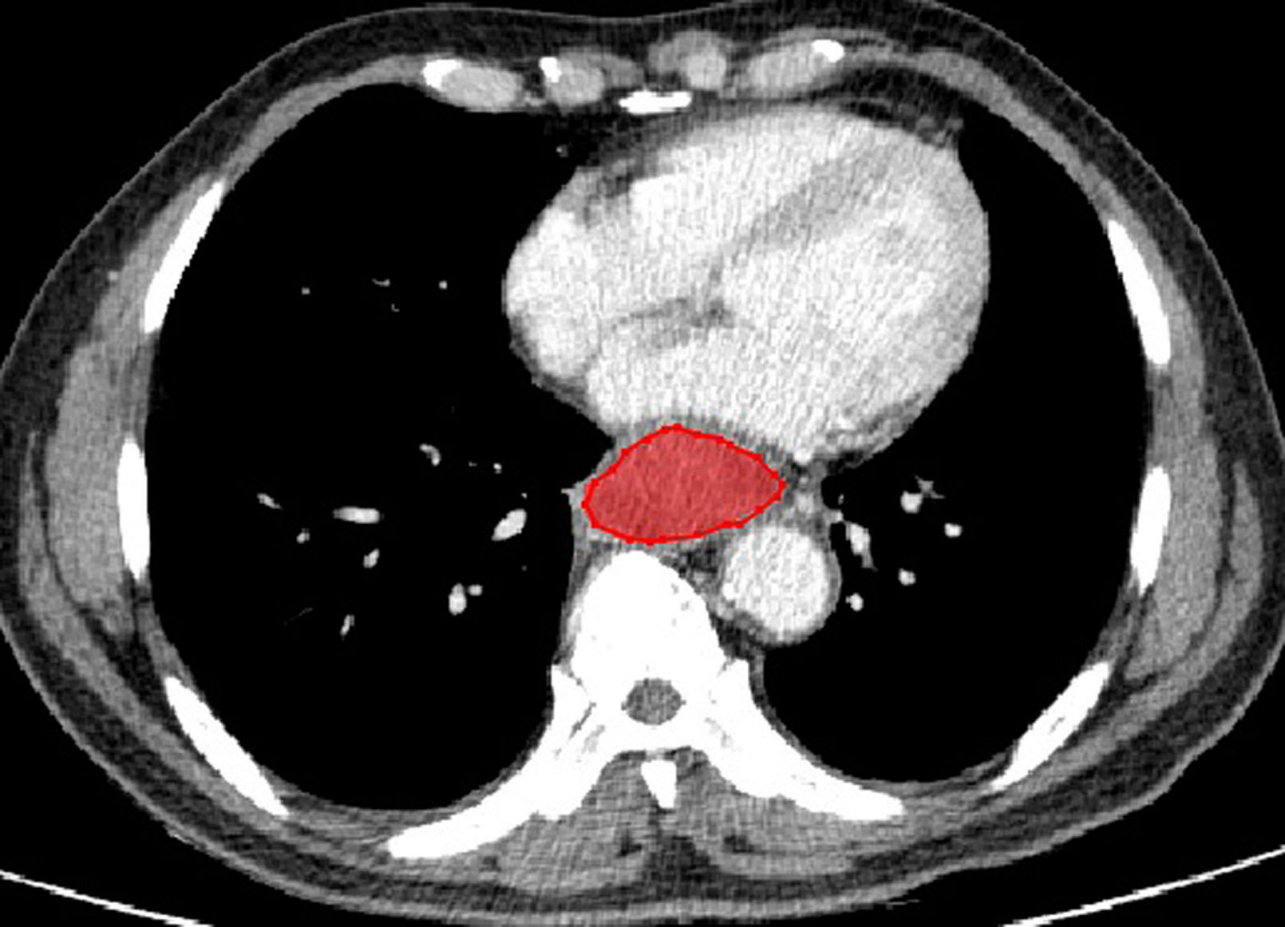
A enhanced CT of a 69-year-old man with esophageal NEC in the middle segment of esophagus (cT3N2M0). There is a moderately enhanced lesion with well-defined margin without necrosis and calcification. The red outline depicts an example of the drawn volume of interest (VOI) covering the largest image slice of the primary lesion.

**Figure 3:**
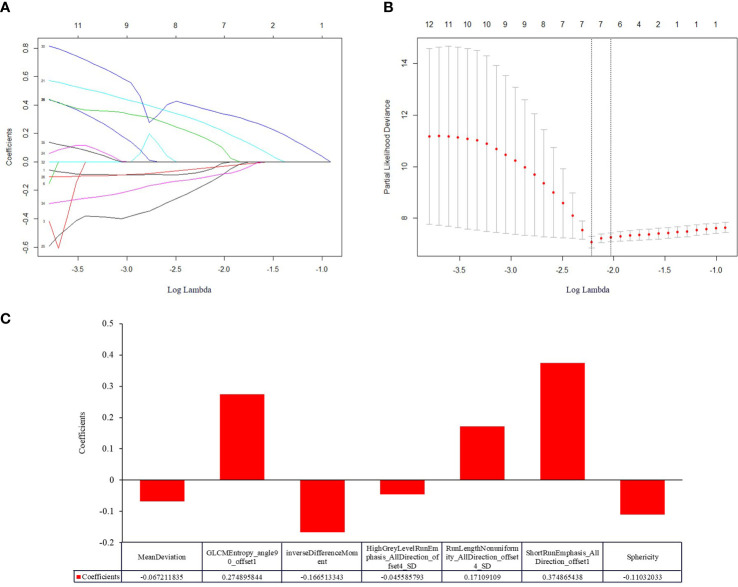
Construction of a radiomic signature predictive of OS in NEC after treatment using the LASSO regression model with a 10-fold cross validation. **(A)** The filtered features derived from the least absolute shrinkage and selection operator (LASSO) regression analysis. The LASSO coefficients of the 36 radiomics features based on entire tumor volumetric in pretreatment venous phase CT images. **(B)** The superparameter lambda (λ) in LASSO was determined by the 10-fold cross-validation. Features with non-zero coefficient were selected for model development. LASSO coefficient analysis of the 36 radiomics features, using 10-fold cross-validation, fourteen nonzero coefficients were selected. **(C)** Histogram displaying seven selected radiomic features that contribute to the constructed signature based on entire tumor volume in the pretreatment venous phase. The x-axis represents the individual radiomic features, with their coefficients in the LASSO Cox analysis plotted on the y-axis.

The typical example of the drawn VOI covering the largest image slice of the primary lesion is depicted in (**Figure 2**). The inter-observer reproducibility analysis was measured by ICC on the VOI-based feature extraction, with a mean ICC of 0.67 (range: -0.03 to 1). The LASSO regression was used for data dimension reduction, feature selection, and radiomics signature building. After feature selection derived from the LASSO regression, the COX regression clinical prediction model was constructed, and the nomogram was created to predict individuals. The result of data dimension reduction with LASSO Cox regression models is described in (**Figures 3A, B**). The seven selected radiomics features consisted of mean deviation, GLCM Entropy_angle90_offset1, inverse difference moment, high Grey Level Run Emphasis_All Direction_offset4_SD, Run Length Nonuniformity_All Direction_offset4_SD, Short Run Emphasis_All Direction_offset1, and Sphericity. The values of the seven selected features in each patient were applied into the formula, and the Rad-scores were obtained to reflect the risk. The Rad-scores thus derived by a combination of the seven most valuable variables and their coefficients are shown in (**Figure 3C**).

### 3.3 Performance and validation of radiomics model

**Figure 4:**
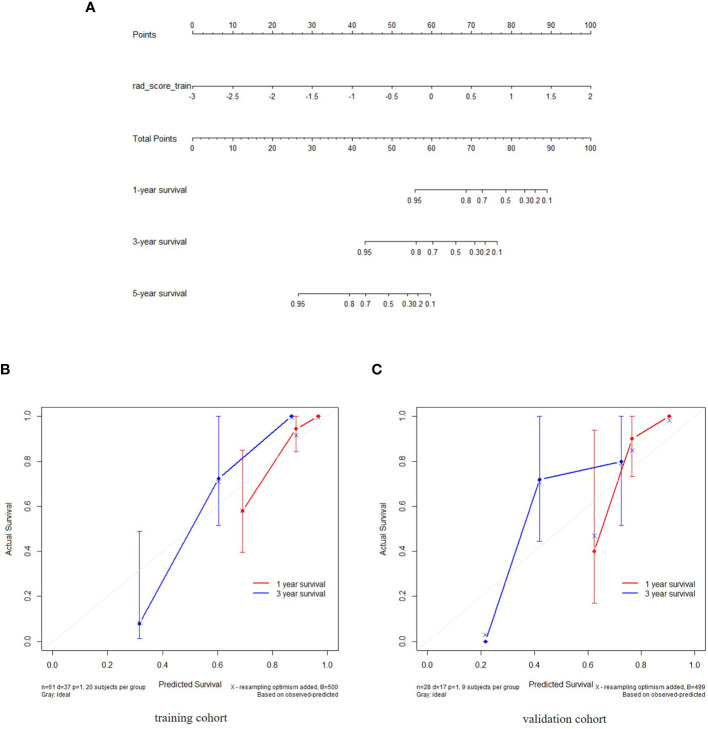
The radiomics nomogram constructed during the venous phases depicting radiomics signature to estimate OS for NEC candidates, along with the assessment of the model calibration. **(A)** The radiomics nomogram was developed using individually predicted OS in NEC after treatment. **(B, C)** Calibration curves for the radiomics nomogram showing the calibration of the model in terms of the 1-year and 3-year survival outcomes in the training and validation datasets. The diagonal dashed line indicates the ideal estimation by a perfect model. The solid line represents the performance of the nomogram. The closeness of the solid line to the diagonal dashed line is an indicator of a better estimation.

For clinical variables, as described in (**Table 2**), the univariate Cox regression analysis indicated that only the enhancement degree was an independent factor that influenced the OS. For the combined model with both radiomics features and clinical variables, the combined score was calculated using selected features and variables. The nomograms for radiomics-based model and the combined model were built based on the training set. One-year, 3-year and 5-year survival probabilities estimated with the nomograms, indicated a good fit for both models in the training and testing sets as seen in (**Figure 4**).

**Table 2:** The univariate regression analysis to identify prognostic factors for overall survival in training cohort.

Variable	Hazard Ratio	95% CI	*P*
Gender	0.868	0.5864-2.266	0.68
Age	1.008	0.9553-1.031	0.698
Morphological subtype	0.943	0.803-1.4	0.679
Tumor length (mm)	1.004	0.980-1.013	0.678
Tumor location	0.586	0.955-3.049	0.071
Clinical symptom	1.004	0.876-1.132	0.948
Depth of wall thickness (mm)	1.012	0.957-1.02	0.47
Ki-67 (%)	0.089	0.004-1.683	0.107
T stage	0.971	0.587-1.808	0.917
N stage	1.131	0.558-1.401	0.6
M stage	1.524	0.304-1.414	0.282
Treatment strategy	1.251	0.466-1.371	0.416
Tumor margin	0.919	0.373-3.165	0.878
Calcification	3.859	0.034-1.995	0.195
Enhanced homogeneity	1.462	0.236-1.979	0.484
Enhanced degree	2.413	0.246-0.697	<0.001*

* Statistically significant.

**Figure 5:**
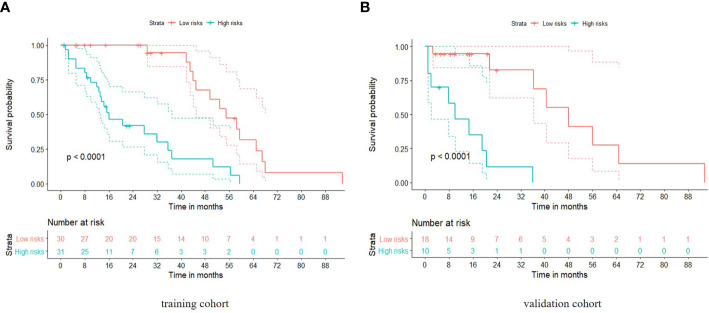
Kaplan-Meier analyses of overall survival based on the radiomics model with cut-off values at the median of the training and validation datasets. The solid line represents the survival curve, while the dotted line stands for the 95% confidence interval. **(A)** Survival curve in the training cohort; **(B)** Survival curve in the validation cohort.

Patients were divided into high and low risk groups using the predicted risk of the model. For the radiomics based model and the combined model, the thresholds were adopted as the median values of the distribution of the risk groups for OS in both, training and testing datasets. To find the best threshold to make the best classification in radiomics based model, the highest value of the Youden index is selected. Threshold values were -0.234 for the radiomics based model. As depicted in (**Table 3**), the radiomics signature based model showed a favorable predictive efficacy than the combined model that integrated the radiomics signature with clinical risk factor, with a C-index of 0.844 (95% CI: 0.783 - 0.905) and 0.847 (95% CI: 0.782 - 0.912) in the training set, and a C-index of 0.805 (95% CI: 0.707 - 0.903) and 0.745 (95% CI: 0.639 - 0.851) in the testing set, respectively. The difference was statistically significant in the training and testing datasets (*p* < 0.0001 for each comparison). The corresponding Kaplan-Meier curves are shown in (**Figure 5**).

**Table 3:** Comparison of predictive performance of the different models for OS in training and testing cohort.

Model	Training cohort C-index (95%CI)	Testing cohort C-index (95%CI)
R model	0.844 (0.905-0.783)	0.805 (0.903-0.707)
R-C model	0.847 (0.912-0.782)	0.745 (0.851-0.639)

R model, Radiomics model; R-C model, Radiomics-clinical model; CI confidence interval.

## 4 Discussion

We designed the present study to explore the application of pretreatment CT radiomics as a significant prognostic biomarker for the overall survival of patients with esophageal NEC. Our results demonstrated that the radiomics signature from the texture features showed improved predictive ability than the combined model incorporating enhanced CT morphological feature and texture features in the venous phase. In addition, the radiomics nomogram integrating the radiomics signature had a significantly improved ability to predict the overall survival than that of the combined nomogram based on clinical and texture factors.

Factors affecting tumor heterogeneity such as tumor phenotype, biological morphology, gene phenotype and other aspects, were demonstrated to be significantly contributory to the development of tumors. Several studies have shown that radiomics plays a critical role in medical decision making by quantitatively assessing the heterogeneity within tumors (18, 24). This study explored better predictors of survival for patients with NEC and intended to construct a radiomics model for patient survival. It was observed that the radiomics model reflected both, the information regarding intra-tumoral heterogeneity as well as its CT enhancement characteristics.

In the present study, seven radiomics features were identified that posed as significant variables in the radiomics score model. These features comprised four categories. One feature included histogram-based analysis that enabled quantification of the distribution of CT values. This tended to be intuitive and sensitive in distinguishing the heterogeneity within the tumor and between tumors. As reported in previous studies, histogram textural features were related to heterogeneity and morphologic characteristics (25–27). Two features derived from GLCM reflected the frequency of the CT values in a voxel, that in turn occurs in a specific spatial relationship with an adjacent voxel (26). Ganeshan et al. demonstrated that patients who had heterogeneous tumors with alterations in GLCM textural features (e.g. high entropy values) demonstrated poorer survival for esophageal cancer (27). Considering the gray range matrix of a two-dimensional image and the characteristic statistics, three GLRLM based features, were calculated to produce considerably better results in distinguishing tumor heterogeneity (28). A formfactor feature named ‘sphericity’ described the texture by identifying the shape, size and specification of each voxel. Kim et al. found that an irregular tumor boundary could be a sign of poor survival (29). Therefore, the findings in this study were in line with previous analyses, in which CT imaging features exhibited better performance in discrimination of heterogeneity and prediction of prognosis.

Owing to its rarity, tumor heterogeneity, nonspecific presentation, unique indolent biology, as well as insufficient awareness, NEC often presents with poor differentiation, a large tumor volume and a deep invasion depth (9, 10). Esophageal NEC is usually depicted to have characteristics of a high-grade malignancy with poor differentiation, inadequate tumor vascularization, and easy metastasis (9). In the present study, there were differences in tumor blood supply and degree of enhancement on CT in the univariate Cox regression analysis. Hence, it was inferred that the differences in blood supply and enhancement degree are likely to influence the biological behavior, growth rate, invasiveness, sensitivity to medicine, and the survival prognosis of the tumor.

In terms of prognostic nomogram analysis for esophageal NEC, Zhang et al. found that the nomogram derived from clinical survival analysis was useful for risk stratification of mortality in patients with esophageal NEC (10). The decision curve analysis ranged from 0.268 to 0.968 for the threshold probability, with a C-index of 0.723. However, the results of Zheng et al. originated from purely large clinical data. This was different from our study which included basic image parameters involved in the clinical model, such as cTNM, tumor homogeneity, enhanced degree, etc. In our study, the radiomics based model exhibited a difference between the high and low-risk patients with respect to overall survival, compared to the combined model. This result was slightly different from the previous studies in cervical cancer by Wang et al. (30), in studies in lung cancer by Coroller et al. (31), and those in oropharyngeal carcinoma by Leijenaar et al. (32). The aforementioned investigators reported better performance of the combined model integrated with clinical and texture features. It was speculated that this difference is primarily result from the uncharacteristic imaging manifestation in esophageal NEC, which may influence the diagnostic value when the conventional CT enhancement features of the entire tumor are observed. Moreover, the bias in the measurement of clinical factors is closely related to insufficient subjects in each cohort. Furthermore, the enhanced level of the tumor was set based on the enhancement degree contrast to the adjacent muscles. However, this is likely to be inevitably influenced by subjective factors based on the observer and the contrast agent distribution in individuals. Therefore, it was concluded that the combined model mildly lowers the diagnostic efficiency for predicting the OS in patients with esophageal NEC.

### 4.1 Limitations

This study has several limitations. Firstly, this is a retrospective study with a small sample size, which might have caused instability in features selection in both, the training and the testing sets. Future studies with larger samples are needed to thoroughly validate the results of this study. Secondly, the results were derived from the use of two CT scanners with a different number of detectors, which might have contributed to a bias in radiomics features extraction. Moreover, additional imaging modalities, such as radiomics features derived from pretreatment functional MR images can provide information on different aspects of tumor characteristics that can be crucial for predicting tumor prognosis. Future research in this context will aim at the correlation between the imaging features and gene or protein biomarkers for survival prediction.

## 5 Conclusion

A practical radiomics model was developed using pretreatment CT radiomics features to predict the overall survival in patients with esophageal NEC. This model incorporated textural features containing effective and reliable prognostic information to stratify patients into statistically significant risk groups.

## Data availability statement

The original contributions presented in the study are included in the article/**Supplementary Material**. Further inquiries can be directed to the corresponding author.

## Ethics statement

This study was approved by the Institutional Review Board (IRB) of the First affiliated hospital of Zhengzhou university. The patients/participants provided their written informed consent to participate in this study. Written informed consent was obtained from the individual(s) for the publication of any potentially identifiable images or data included in this article.

## Author contributions

JG and YZ designed the research. YZ, LS and JX performed the research and data analysis. HL contributed to the CT radiomics analysis and statistical analysis. YZ and JJX collected the data and assigned the forms. YZ, HL and JG wrote the paper. All authors contributed to the article and approved the submitted version.
